# Temporal Dynamics of Early Somatosensory Processing in Goal-Directed Actions

**DOI:** 10.1523/JNEUROSCI.0349-26.2026

**Published:** 2026-10-06

**Authors:** Elena Fuehrer, Dimitris Voudouris, Lisa K. Maurer, Katja Fiehler

**Affiliations:** ^1^Justus Liebig University Giessen, Giessen 35390, Hesse, Germany; ^2^Center for Mind, Brain and Behavior, Universities of Marburg, Giessen and Darmstadt, Marburg 35032, Hesse, Germany

**Keywords:** reaching, sensorimotor prediction, sensory gating, somatosensory evoked potentials, tactile suppression, task relevance

## Abstract

Tactile sensitivity is temporally modulated on a moving limb. Yet the neural mechanisms underlying this modulation remain unclear. Using electroencephalography, we examined the temporal dynamics of early somatosensory processing during goal-directed reaching. Participants (self-reported gender, 22 females, 16 males) reached for their unseen left hand while a brief standard vibration of a fixed intensity was applied to the moving right index finger in different movement phases. After each reach, participants compared this standard with a second comparison vibration of varying intensity applied to the sternum and judged which vibration felt stronger. The same task was performed at rest to account for individual differences. Psychophysical results revealed an overall reduction in tactile sensitivity during reaching compared with rest, with sensitivity transiently improving around maximum speed relative to the late movement phase. Consistent with the psychophysical data, the short-latency P45 component, originating in the primary somatosensory cortex, was reduced during reaching but recovered around maximum speed. Lower P45 amplitudes were associated with lower tactile sensitivity measured behaviorally, suggesting that early somatosensory processing may contribute to the observed perceptual changes. This temporally specific modulation of tactile perception, together with corresponding changes at the early cortical stage, is consistent with the idea that the somatosensory system dynamically adapts its sensitivity to facilitate control of somatosensory-guided actions.

## Significance Statement

The perception of touch on a moving hand varies across movement phases, depending on the importance of somatosensory feedback for guiding the hand toward the movement target. Our results show that these changes in perception are associated with modulations in early brain activity. This indicates that the central nervous system adjusts touch processing in a time-sensitive manner at early processing stages, which may support goal-directed actions guided by somatosensory feedback.

## Introduction

Goal-directed movements depend on somatosensory signals. Tactile sensations arise directly from physical interactions involving the moving limb, and proprioceptive and tactile feedback inform about limb position and movement dynamics. Some somatosensory signals provide essential information for sensorimotor control (Goulding et al., 2025), whereas others may be irrelevant. Because feedback-based mechanisms are noisy (Faisal et al., 2008) and delayed (Miall and Wolpert, 1996), the sensorimotor system likely integrates sensory feedback with predictive feedforward models using efference copies of motor commands to estimate future sensory states of the moving limb (Desmurget and Grafton, 2000).

A well-documented sensorimotor phenomenon is a reduction in tactile sensitivity on the moving limb, known as tactile suppression (Chapman et al., 1987). This phenomenon has been demonstrated behaviorally across a wide range of actions and is complemented by electroencephalographic (EEG) findings, showing gating (reduced amplitudes) of somatosensory evoked potentials (SEPs) during movement. This gating is evident in short-latency components like the N20 and P45 (Rushton et al., 1981; Rossini et al., 1999; Lei et al., 2018), which reflect early-stage processing in the primary somatosensory cortex (S1; Allison et al., 1992).

Crucially, tactile suppression is not an all-or-nothing phenomenon: When sensory input from the moving limb is relevant for the movement, tactile sensitivity is partly preserved, although some suppression may persist. For example, tactile suppression is less pronounced on the index finger during a precision grip than on other parts of the hand and arm (Colino et al., 2014; Manzone et al., 2018) or when grasping a slippery versus a high-friction object without vision (Voudouris and Fiehler, 2022). Tactile sensitivity also recovers on the lower leg shortly before perturbations during a balancing task (Wachsmann et al., 2025b). Likewise, tactile suppression is modulated during targeted reaching without vision, relying exclusively on somatosensory feedback, and is transiently released around maximum speed (Voudouris and Fiehler, 2021), i.e., the onset of the deceleration phase when sensory guidance becomes important (Elliott et al., 2001). Together, these behavioral findings indicate that tactile suppression may be flexibly modulated by the relevance of somatosensory feedback, with reduced suppression reflecting a facilitation of task-relevant input.

Electrophysiological studies likewise show that task-relevant feedback from the moving limb is modulated at early stages of somatosensory processing: short-latency SEP amplitudes increase during exploratory hand movements compared with rest (Knecht et al., 1993) and during lower limb movements in a balancing task when an upcoming perturbation challenges posture (McIlroy et al., 2003). Similarly, the somatosensory evoked field component M38 is enhanced during grasping relative to rest and further increased during a more complex motor task (Wasaka et al., 2017). Together, these findings suggest an enhancement of early somatosensory processing (i.e., weaker SEP gating) when feedback is relevant for action control. However, because previous studies have typically focused on either tactile perception or neural responses, the relationship between them remains unclear. Moreover, prior work has largely compared discrete task conditions, leaving open how tactile perception and cortical somatosensory processing are dynamically coupled as feedback demands change.

Here, we combined psychophysics with EEG recordings of SEPs to test whether dynamic changes in tactile suppression are reflected in early somatosensory processing during a somatosensory-guided reaching task previously shown to modulate tactile sensitivity (Voudouris and Fiehler, 2021). First, we asked whether tactile suppression is modulated across different movement phases; second, whether early cortical processing reflected in the P45 component shows a corresponding temporal modulation; and third, whether temporal changes in tactile sensitivity are associated with cortical changes. Based on prior evidence (Rushton et al., 1981; Rossini et al., 1999), we predicted that movement-related tactile suppression (i.e., lower tactile sensitivity) would correspond to reduced early somatosensory processing, as indexed by the P45 component, and that P45 amplitudes would mirror the temporal dynamics of suppression across movement phases. Since perceptual reports also depend on higher-order processes, such as stimulus evaluation and decision-making (Heekeren et al., 2008), we additionally examined whether the later P300 showed a similar temporal modulation.

## Materials and Methods

### Participants

Thirty-eight participants (self-reported gender, 22 females, 16 males; mean age, 25.7 years; SD = 4.1) provided written informed consent and received either course credit or 10 €/hour as compensation. The study was approved by the ethics committee of the Justus Liebig University Giessen and conducted in accordance with the Declaration of Helsinki (World Medical Association, 2013), with the exception of preregistration. All participants were right-handed, as assessed by the Edinburgh Handedness Inventory (Oldfield, 1971; *M* = 90.8, range = 28.6–100). Two participants were excluded for slow movements, leaving too few stimulus presentations in the late post-max speed phase to fit psychometric functions (see below, Kinematic analysis); one participant was excluded because 86% of trials were rejected (see below, Kinematic analysis); one participant was excluded as a statistical outlier (see below, Statistical analysis).

### Experimental design

Vibrations (20 ms, 280 Hz) were delivered using a vibrotactile stimulation device (PiezoTac tactor; Engineering Acoustics). The tactor comprised a skin contactor (6.4 mm diameter) mounted on a shielded cantilever. Cantilever vibrations produced sinusoidal oscillations perpendicular to the skin. One contactor was placed on the palmar side of the proximal phalanx of the right index finger, leaving the fingertip uncovered. A second contactor was positioned on the sternum, ∼3 cm below the clavicle. The contactors were fixed to the participant's skin using a medical tape. The experiment was controlled by the custom MATLAB (The MathWorks) software using the Psychtoolbox extension (Brainard, 1997). The position of an infrared marker on the nail of the right index finger was recorded at 250 Hz with an Optotrak Certus motion-tracking system (Northern Digital).

### Procedure

Participants placed both hands on a table in front of them. The left hand rested palm-down with the thumb and index finger on specific markers positioned 10 cm to the left of the body midline to ensure consistent placement. The right hand rested on a keypad located 18 cm to the right of the midline and 20 cm in front of the participant. Throughout the experiment, participants' view of their hands was occluded using a tilted cardboard screen, which allowed them to see the top of the computer monitor for instructions. At the beginning of each trial, participants held down a key with their right index finger to initiate the trial. Text on the computer screen then indicated the target finger on the left hand (thumb or index), and participants had 2,500 ms to initiate the movement. Upon releasing the key, participants performed a reaching movement with the right hand to touch the nail of the specified target finger. A standard vibration (peak-to-peak displacement, 119 µm) was delivered to the moving right index finger at one of five possible latencies relative to movement onset (50, 150, 250, 350, or 450 ms), which was determined by the key release. After reaching the target, participants rested their right hand next to the left hand on the table. A comparison vibration was delivered to the sternum 3,000 ms after the standard vibration, well after movement completion, while both hands were still. The comparison stimulus had one of seven possible intensities (peak-to-peak displacement, 47, 71, 95, 119, 143, 167, or 192 µm). Participants indicated which of the two vibrations felt more intense by pressing foot pedals: the left pedal for the first vibration (on the hand) and the right pedal for the second vibration (on the sternum; see Fig. 1 for the trial sequence). Each stimulation time was presented 70 times for a total of 350 trials. Additionally, 70 trials (16.67% of all trials) were included to record movement-related EEG without vibrotactile stimulation, matching the number of trials for each stimulation time. In these omission trials, participants were informed that no vibration was given 3,000 ms after movement onset and no tactile discrimination response was required. Participants completed 10 blocks of 42 trials each. Within each block, all combinations of stimulation time and comparison intensity were presented once, along with seven omission trials in a random order. Trials were repeated at the end of the block if participants initiated the movement too early, <180 ms after target presentation, suggesting the cue had not been processed or if they failed to initiate movement within 2,500 ms after the cue. Self-paced breaks were provided between blocks. Before starting the experiment, participants familiarized themselves with the reaching movement and the discrimination task in 20–40 practice trials.

**Figure 1.**
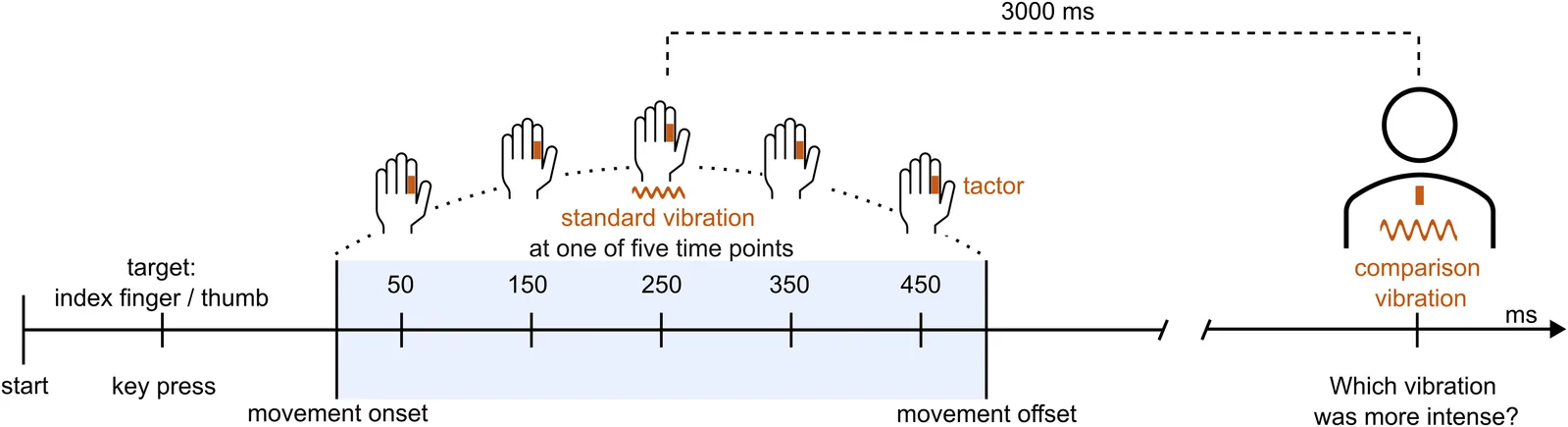
Trial sequence. Participants initiated each trial by pressing and holding a key with the right index finger. Text on the monitor indicated the target finger of the left hand. Participants released the key and reached toward the target finger of the left hand. A standard vibration of fixed amplitude was delivered to the right index finger at one of five time points during the movement (250 ms shown here). After an ISI of 3,000 ms, a comparison vibration of varying amplitude was presented on the sternum. Participants judged which vibration felt stronger using foot pedals. Participants also performed the same psychophysical task with both hands at rest.

We also assessed tactile sensitivity at rest by asking participants to perform the discrimination task without movement, while both hands remained on the table in the same position adopted after the reaching movements. This enabled us to assess relative changes between rest and movement phases and determine the effect of movement on tactile processing while accounting for idiosyncrasies in tactile sensitivity. The standard vibration was presented to the right index finger at one of five delays (50, 150, 250, 350, or 450 ms) after a fixed interval of 700 ms from trial onset, replicating the relative timing used during movement. A comparison vibration followed on the sternum 3,000 ms later. Participants judged vibration intensity using foot pedals. Each of the seven comparison intensities was presented twice per stimulation time (70 trials total). Trials ended only after participants had responded and at least 6,000 ms had elapsed from trial onset, ensuring a trial duration consistent with the reaching block. Trials started automatically after a foot pedal was pressed. The rest condition was divided into two blocks, separated by a short break, and both blocks were administered either before or after the movement blocks, counterbalanced across participants.

### Kinematic analysis

The kinematic data were analyzed in MATLAB (R2021a; The MathWorks). Movement speed was computed by numerical differentiation of the three-dimensional data divided by the sampling interval of 4 ms. Movement onset was defined as the key release. Mean reaction time was 547 ± 240 ms (standard deviation across participants). Movement offset, i.e., when participants touched the target finger, was determined using the multiple sources of information method to form a composite score (Schot et al., 2010): coarse spatial constraints excluded frames with lateral positions near the start zone (all positions within 20 cm to the left and 10 cm to the right of the start position were excluded). To lower the likelihood of a frame after touching the target finger representing the end of the movement, frames were down-weighted when the moving index finger was within 3 cm of the location on the table where participants rested their hand after the touch. A timing gate increased the likelihood of a frame being the end of the movement once speed fell below 0.10 m/s after the speed maximum (identified within 400 ms following movement onset) and maintained this elevated score for 80 ms. Lower instantaneous speed, smaller lateral positions (toward the target hand), and larger sagittal positions (further from the body) also increased the score. The movement end was defined as the frame with the highest composite score likelihood.

Movement duration (*M* = 562 ms, SD = 100 ms) was defined as the time between movement onset and offset. Time of maximum movement speed was determined for each trial. To account for interindividual differences in movement duration, the timing of maximum speed was normalized to the total movement duration in each trial. Across participants, the average maximum speed was 1.22 ± 0.19 m/s and occurred after 202 ± 62 ms, which corresponded to 33 ± 6% of the movement. Because maximum speed denotes the onset of the period when sensory guidance of the hand toward the target gains importance (Elliott et al., 2001), stimulation trials were assigned to four movement phases defined relative to maximum speed: (1) pre-max speed; (2) window of ±5% around max speed; (3) early post-max speed, the first 40% of the remaining post-max duration; and (4) late post-max speed, the final 60% of the remaining post-max duration. These time bins will be referred to as the four movement phases. We used a 40/60 rather than 50/50 proportion to divide the post-max time to balance trial counts: The average movement duration was 562 ms, while the latest stimulation occurred 450 ms after movement onset, which resulted in too few late-phase trials for stable psychometric fits under a 50/50 post-max speed split in eight participants. The 40/60 proportion allowed for stable psychometric fits in seven of these eight participants. Critically, the 40/60 split also yielded more balanced trial counts per movement phase, with Phases 1–4 containing on average 32.9, 13.7, 27.1, and 26.3% of trials, respectively. We allowed fewer trials in the small window around maximum speed to maintain temporal specificity in this movement phase.

Trials were excluded from all further analyses if the movement end could not be determined (empty composite score; <1% of trials); if >25% of motion-tracking samples were missing in the last third of the movement due to marker occlusion (1% of trials); if the mean speed during the first 20% of the movement was below 0.1 m/s (1% of trials), indicating delayed movement initiation after start key release and incorrectly timed tactile stimulations; if the movement duration was >3 SD above the participant's within-block mean (1% of trials); or if the stimulation occurred after the movement had ended (4% of trials).

### Psychophysics analysis

Logistic psychometric functions were fitted to participants' responses in the psychophysical task using the maximum likelihood estimation implemented in the Psignifit 4 toolbox (Schütt et al., 2016) in MATLAB. Separate functions were fitted for each participant, both for the four movement phases and for the resting condition. Across participants, the mean number of trials included in the rest condition, pre-max, around max, early post-max, and late post-max phases was 70, 108, 45, 88, and 83, respectively. PSEs were derived from the 50% point of each function. To control for interindividual differences in tactile intensity discrimination, the PSE in each of the four movement phases was subtracted from the corresponding values in the rest condition to compute four normalized difference scores (PSE_diff_) per participant (Fig. 2).

**Figure 2.**
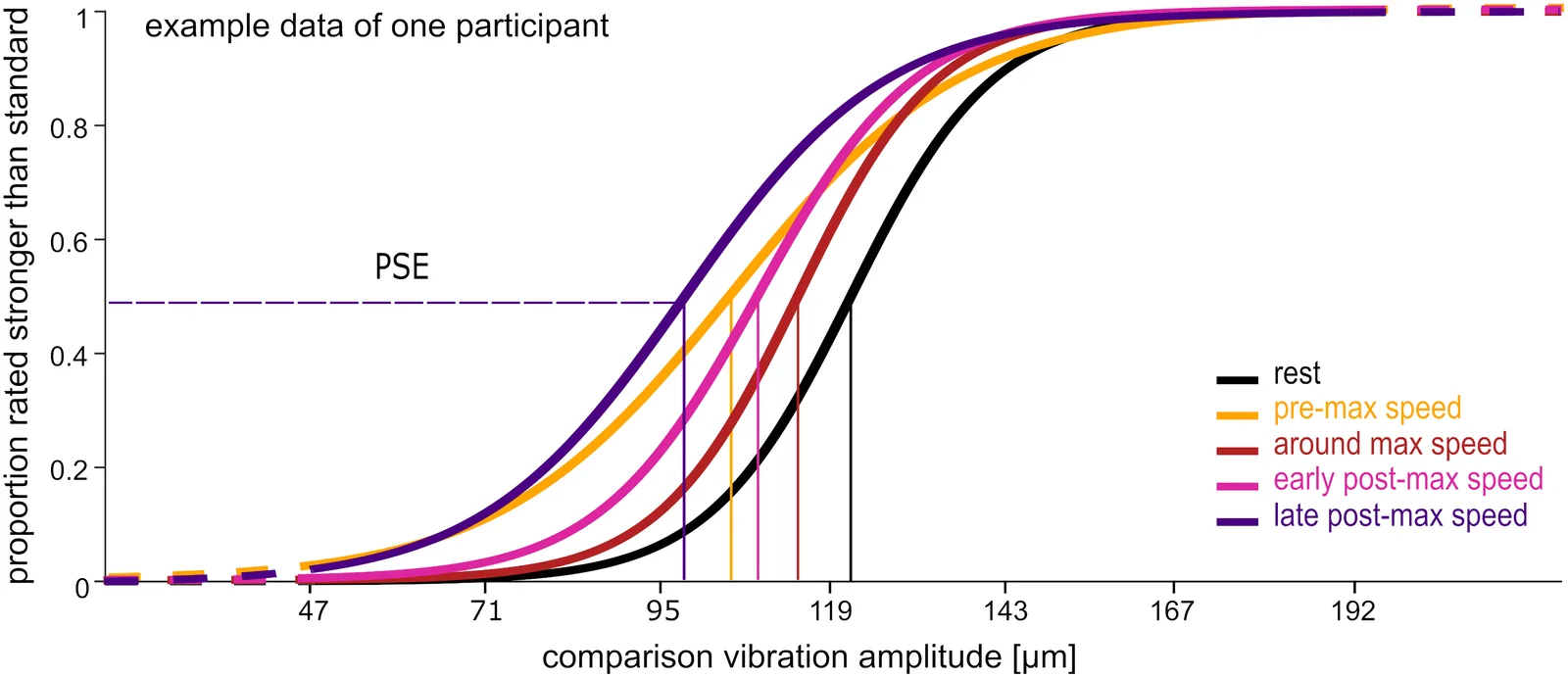
Psychometric curves of one example participant. Psychometric curves were fitted to the participant's responses in the comparison task, both for the rest condition and for each movement phase. The PSE was determined as the 50% point of each curve. To control for individual differences in tactile sensitivity, the PSE from each movement phase was subtracted from the PSE in the rest condition, yielding one difference score per movement phase (PSE_diff_).

### EEG analysis

EEG data were recorded from 37 channels (Fp1, C3, P3, P4, C4, AF7, AF3, AFz, F1, F5, FT7, FC3, C1, C5, TP7, CP3, P1, P5, PO7, PO3, POz, PO4, PO8, P6, P2, CPz, CP4, TP8, C6, C2, FC4, FT8, F6, AF8, AF4, F2, FCz) placed according to the international 10–20 system, using a BrainProducts actiCap with a 64-channel LiveAmp system and BrainVision Recorder software (Brain Products), sampled at 500 Hz. In addition, electrodes were placed at the outer canthi of both eyes and below the left eye to record horizontal and vertical eye movements. Data were processed using EEGLAB 2024.2 (Delorme and Makeig, 2004) in MATLAB. Preprocessing involved high-pass filtering (noncausal Butterworth impulse response, half-amplitude cutoff at 0.1 Hz, 12 dB/octave roll-off), removal of DC offsets, and attenuation of 50 Hz line noise using Zapline-plus (Klug and Kloosterman, 2022). Data were rereferenced offline to the average of the linked earlobes. Noisy channels were identified through visual inspection and interpolated. Independent component analysis (ICA) was performed using the Infomax algorithm, with PCA rank reduction used when channels had been interpolated. Ocular artifacts were removed by ICA and ICLabel classification (Pion-Tonachini et al., 2019). Remaining artifacts were rejected via artifact subspace reconstruction. Stimulation triggers were latency-corrected by 4 ms as determined in a dedicated test run of the experiment using a StimTrak system (Brain Products) with an audio adapter and a contact microphone attached to the stimulator's skin contactor. Data were low-pass filtered using a noncausal Butterworth filter (40 Hz half-amplitude cutoff, 12 dB/octave roll-off). EEG data were segmented into epochs from −460 to 400 ms relative to the vibration onset at the five possible stimulation time points (50, 150, 250, 350, and 450 ms postmovement onset) and baseline corrected using the 200 ms prestimulus interval. For omission trials, epochs were extracted in the same manner around the five time points (50, 150, 250, 350, and 450 ms postmovement onset) to obtain the EEG activity associated with movement at the corresponding vibration delivery times.

These omission epochs were averaged separately for each time point and subsequently subtracted from the respective stimulation epochs, isolating the SEPs (Fig. 3*A*). The resulting difference waves were grouped into four movement phases by stimulation timing relative to each trial's maximum speed (pre-max, around max, early post-max, late post-max; see above, Kinematic analysis) and averaged within each phase (Fig. 3*B*). Mean trials per participant were 64, 96, 41, 80, and 78 in the rest condition, pre-max speed, around max speed, early post-max speed, and late post-max speed phases, respectively. On average, 61 omission trials were analyzed per person and time point. The analysis focused on CP3 for the P45 and C3 for the P300, corresponding to the focal maxima of these components observed in a previous study using the same vibrotactile stimulation protocol with stimulation delivered to the right index finger (Fuehrer et al., 2025). This selection is also consistent with the typical contralateral centroparietal P45 and central P300 topographies (Yamada, 2014). Components were identified for each participant using their individual average trace across the rest condition and the four movement phases (Fig. S1). Within predefined ranges, positive peaks were located for the P45 (35–55 ms) and P300 (250–350 ms). Measurement windows were set at ±5 ms of peak latency for P45 and ±10 ms for P300. Mean amplitudes were then extracted per participant, rest condition, and movement phase within these windows. Next, we computed difference scores by subtracting the mean component amplitude of each of the four movement phases from the mean component amplitude of the rest condition, resulting in one P45_diff_ and one P300_diff_ score per movement phase. This rest-movement subtraction was chosen to maintain consistency with the computation of the psychophysical measure.

**Figure 3.**
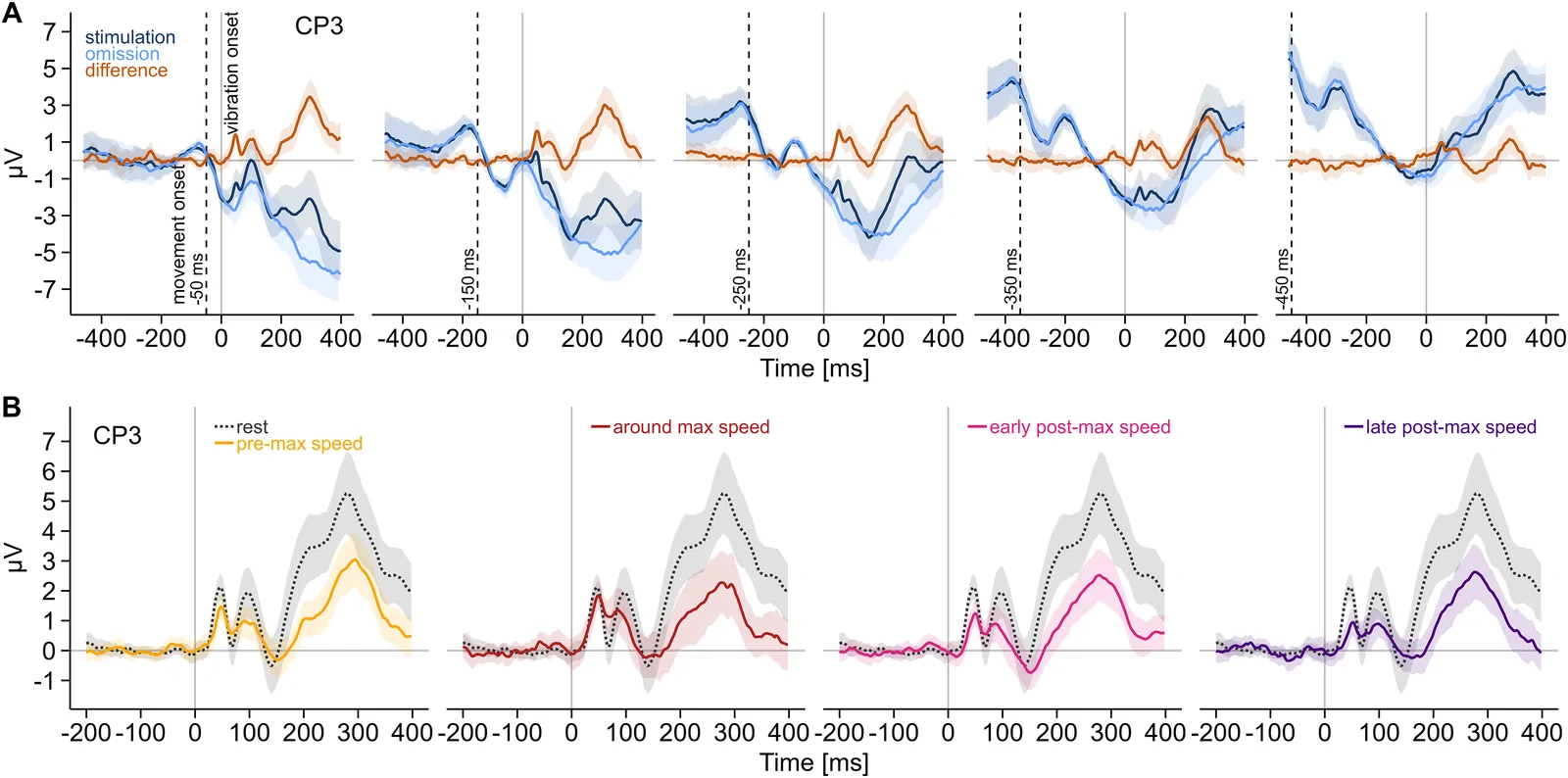
Grand-averaged EEG activity and difference waves across movement phases. ***A***, Grand-averaged EEG responses (*N* = 34) for stimulation and omission trials illustrated at electrode CP3. Stimulation trials with movement and vibrations delivered at five time points (50, 150, 250, 350, 450 ms) postmovement onset. EEG was epoched to vibration onset and baseline corrected to the 200 ms prestimulation interval. Omission trials with movement only, epoched at the same five time points (50, 150, 250, 350, 450 ms) postmovement onset and baseline corrected to the preceding 200 ms. Difference waves computed as stimulation minus omission to isolate SEPs. The dotted vertical line shows movement onset. Omission traces in all subpanels are the same data re-epoched; stimulation traces differ by stimulation time. Shaded bands show 95% CIs. ***B***, Grand-averaged difference-wave SEPs (*N* = 34) grouped into four phases relative to maximum movement speed: pre-max speed (yellow), around max speed (red), early post-max speed (pink), and late post-max speed (purple). Rest condition SEPs, shown for reference (black dotted), are the same in each subpanel. Shaded bands show 95% CIs.

### Statistical analysis

To assess whether movement suppressed vibration perception, we compared the PSE_diff_ from each of the four movement phases with zero using one-sample, two-sided *t* tests. Modulation of tactile perception across the movement time course was tested with a univariate repeated-measures ANOVA on the PSE_diff_ from the four movement phases. Post hoc comparisons used paired-sample *t* tests between movement phases.

To examine modulations in the SEPs associated with movement, we tested P45_diff_ and P300_diff_ from each movement phase against zero using one-sample, two-sided *t* tests. To assess a modulation of component amplitudes across the four movement phases, univariate repeated-measures ANOVAs were performed on P45_diff_ and P300_diff_ separately, followed by paired post hoc *t* tests between movement phases. Planned contrasts that were motivated by significant differences in PSE_diff_ were one-sided; all other comparisons were two-sided. To assess whether SEP amplitudes covaried with changes in tactile perception, a repeated-measures correlation was computed between P45_diff_ and corresponding PSE_diff_ values across movement phases using rmcorr (Bakdash and Marusich, 2017). This approach estimates the common within-participant association across phases while accounting for stable interindividual differences in overall P45_diff_ and PSE_diff_ levels.

Data were analyzed in R, v.4.3.3 (R Core Team, 2024) with a significance level of *α* = 0.05. Greenhouse-Geisser correction was applied when sphericity was violated (Mauchly's test). The false discovery rate (FDR) was controlled within each test family using the Benjamini–Hochberg method, and adjusted *p* values are reported (Benjamini and Hochberg, 1995). Specifically, FDR correction was applied to the PSE_diff_ tests against zero, the PSE_diff_ post hoc phase comparisons, the tests against zero for each EEG component, the ANOVAs across the two EEG components, and the P45_diff_ post hoc phase comparisons. Effect sizes are reported as Cohen's *d_z_* and partial *η*^2^ (Lakens, 2013). Given the final sample size of *N* = 34, a significance level of *α* = 0.05, and 80% power, a sensitivity analysis in G*Power estimated that the smallest detectable effect sizes for the paired-sample post hoc tests were *d_z_* = 0.50 and *d_z_* = 0.44 for two-tailed and one-tailed tests, respectively (Faul et al., 2007). Because FDR correction does not correspond to a single fixed alpha level, this sensitivity analysis should be interpreted as approximate. Univariate outliers were defined as ∣*z*∣ > 3 based on means and standard deviations within movement phases. Bivariate outliers were defined by Mahalanobis distance (*D*^2^) with a cutoff of *χ*^2^_(2, 0.999)_ = 13.82 (Hardin and Rocke, 2005; Finch, 2012). One participant was classified as both a univariate outlier (*z* = −3.63) within P45_diff_ in the around max speed phase and as a bivariate outlier for P45_diff_ and PSE_diff_ (*D*^2^ = 23.09). Data from this participant were excluded from the primary analyses (see Supplement and Fig. S2 for the analysis without this outlier exclusion).

## Results

To account for individual differences in tactile processing, movement-related changes were expressed as difference scores by subtracting PSEs and SEP amplitudes obtained during movement from those measured at rest (PSE_diff_, P45_diff_, P300_diff_; see Materials and Methods). Positive PSE_diff_ values indicate reduced tactile sensitivity during movement relative to rest, i.e., stronger suppression. Positive P45_diff_ and P300_diff_ values reflect smaller SEP amplitudes during movement, i.e., stronger SEP gating. Conversely, smaller difference scores indicate a relative recovery of tactile sensitivity or SEP amplitude relative to rest.

To test tactile suppression during movement, we compared PSE_diff_ from each movement phase to zero using one-sample *t* tests. Tactile sensitivity was reduced relative to rest, with PSE_diff_ significantly greater than zero in all movement phases, indicating tactile suppression: pre-max speed (*t*_(33)_ = 3.47, *p* = 0.002, *d_z_* = 0.59, 95% CI [5.54–21.24]), around max speed (*t*_(33)_ = 2.48, *p* = 0.019, *d_z_* = 0.42, 95% CI [1.73–17.59]), early post-max speed (*t*_(33)_ = 3.59, *p* = 0.002, *d_z_* = 0.62, 95% CI = [6.36–23.01]), and late post-max speed (*t*_(33)_ = 4.20, *p* < 0.001, *d_z_* = 0.72, 95% CI [9.23–26.60]). A repeated-measures ANOVA revealed that tactile suppression varied across movement phases (*F*_(3,99)_ = 4.66, *p* = 0.004, $\eta_{p}^{2}=0.12$). Two-sided post hoc comparisons showed weaker tactile suppression around max speed than in the late post-max phase, i.e., better tactile sensitivity around maximum speed (*t*_(33)_ = −3.3, *p* = 0.014, *d_z_* = −0.57, 95% CI [−13.35– −3.16]), in line with previous findings (Voudouris and Fiehler, 2021). Tactile suppression did not differ significantly between the other movement phases (all *t*_(33)_ ≥ −2.5, *p* ≥ 0.053, *d_z_* ≥ −0.43; Fig. 4*A*).

**Figure 4.**
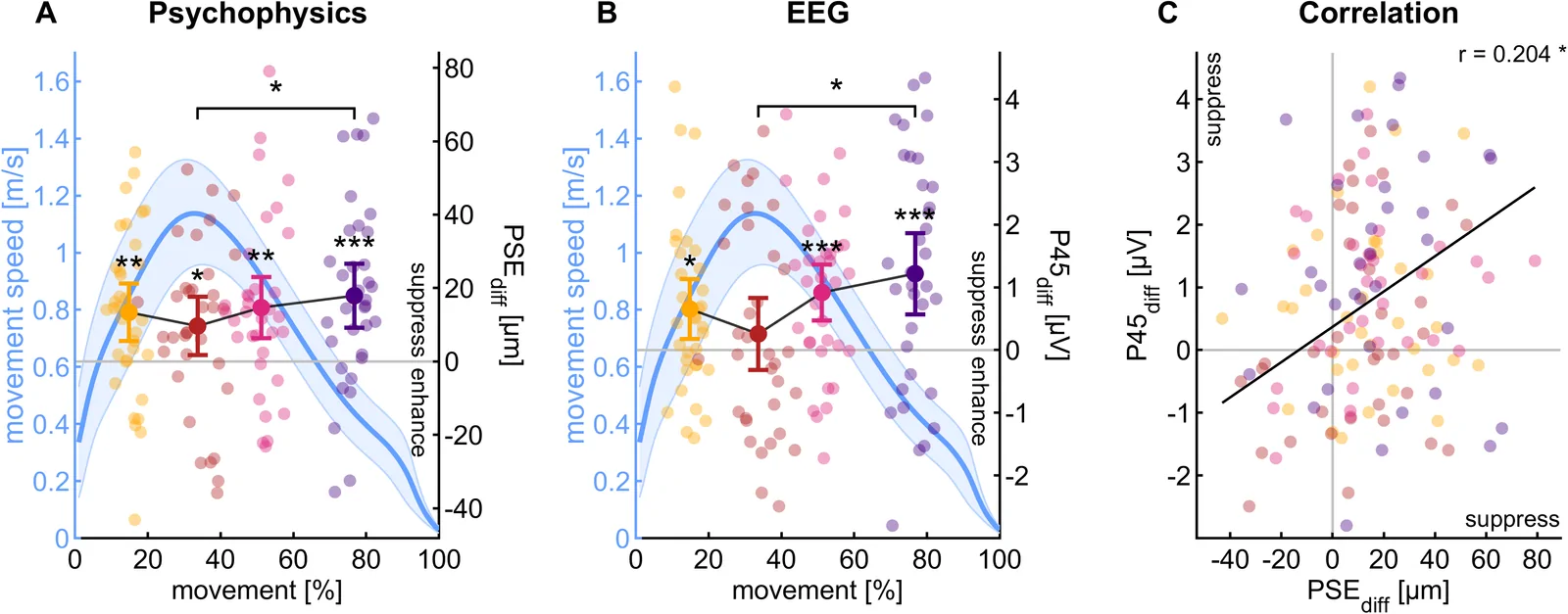
Movement-related suppression in tactile perception and SEP amplitude. Average time-normalized movement speed across participants (*N* = 34; blue line, left *y*-axis). The shaded area indicates standard error. The right *y*-axis shows mean PSE_diff_ (***A***) and P45_diff_ (***B***) for the four movement phases: pre-max speed (yellow), around max speed (red), early post-max speed (pink), and late post-max speed (purple). Error bars indicate 95% confidence intervals. Dots illustrate individual participants. ***A***, Tactile perception was suppressed in all four movement phases (*t* tests against zero). Tactile suppression was weaker around max speed relative to the late post-max speed phase (univariate ANOVA, paired *t* tests). ***B***, P45 amplitude was comparable to rest around max speed and suppressed in all other movement phases (*t* test against zero). P45_diff_ was smaller around max speed than in the late post-max speed phase, indicating less SEP gating mid-reach relative to later in the movement (univariate ANOVA, paired *t* tests). ***C***, Scatterplot of P45_diff_ as a function of PSE_diff_. Each point represents one participant in one movement phase. The black line shows the common within-participant slope from the repeated-measures correlation across all observations plotted through the grand mean. Stronger tactile suppression during movement was significantly associated with lower P45 amplitude (repeated-measures correlation). *p < 0.05, **p < 0.01, ***p < 0.001.

We focused the P45 analysis on electrode CP3 located over S1 contralateral to the stimulated right hand, because P45 amplitude is maximal at this site (Yamada, 2014; Fuehrer et al., 2025). Figure 5*A* shows the waveforms, and Figure 5*C* shows the respective scalp maps across movement phases. To assess P45 gating during movement, we tested P45_diff_ against zero with one-sample *t* tests. P45_diff_ was greater than zero, indicating P45 gating, for most movement phases: pre-max speed (*t*_(33)_ = 2.78, *p* = 0.010, *d_z_* = 0.48, 95% CI [0.18–1.13]), early post-max speed (*t*_(33)_ = 4.18, *p* < 0.001, *d_z_* = 0.72, 95% CI [0.47–1.36]), and late post-max speed (*t*_(33)_ = 3.83, *p* < 0.001, *d_z_* = 0.66, 95% CI [0.57–1.87]). In contrast, P45_diff_ was not significantly different from zero around maximum speed (*t*_(33)_ = 0.91, *p* = 0.367, *d_z_* = 0.16, 95% CI [−0.32–0.83]), indicating a transient recovery of P45 amplitude toward rest around the onset of reach deceleration. Thus, P45 gating was present during most movement phases but was reduced around maximum speed, when somatosensory guidance of the hand gained importance.

**Figure 5.**
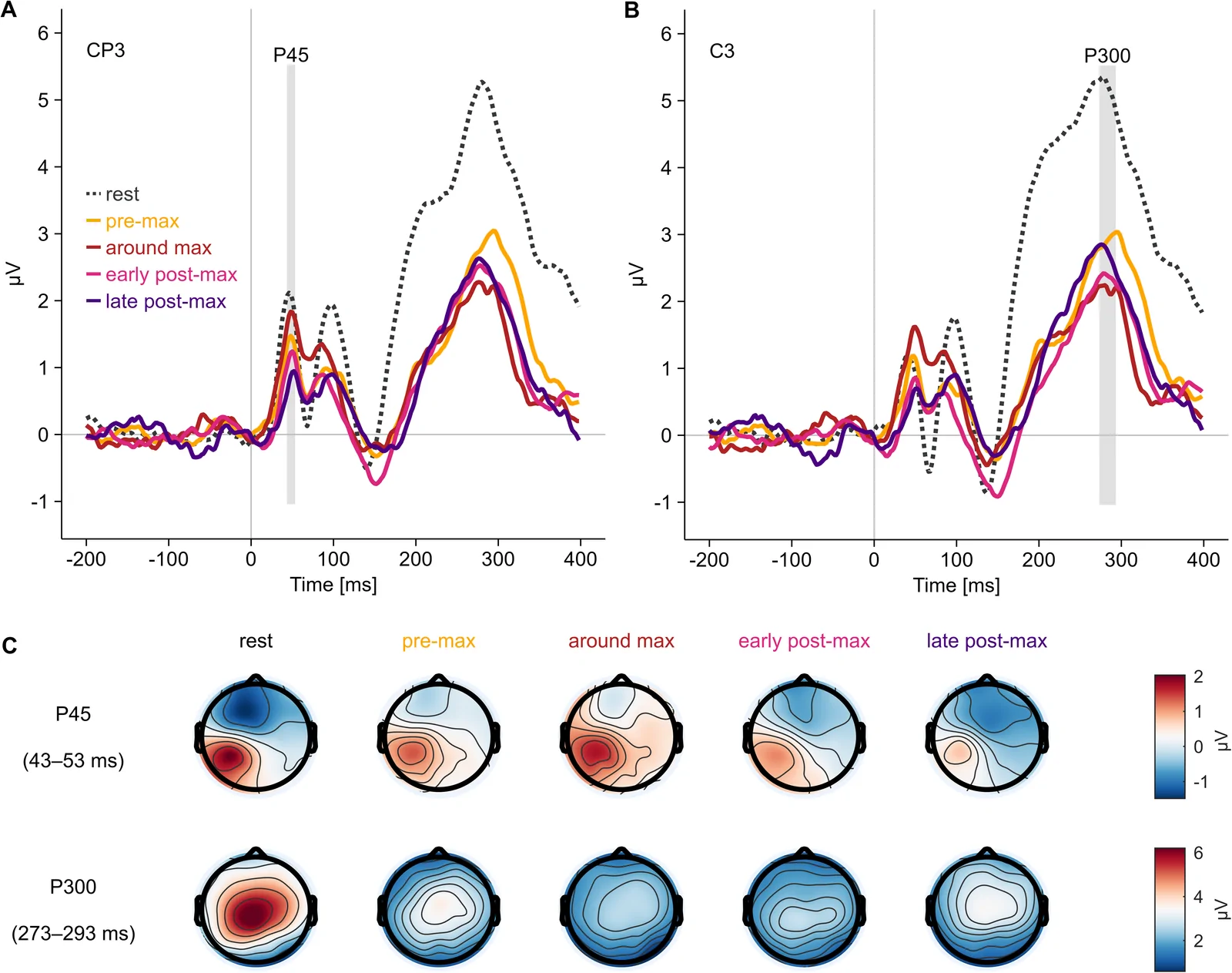
Grand-averaged SEPs during rest and movement. ***A***, Grand-averaged SEPs at CP3 (*N* = 34), time-locked to standard vibration onset, with the P45 highlighted. ***B***, Grand-averaged SEPs at C3 (*N* = 34), time-locked to standard vibration onset, with the P300 highlighted. ***C***, Scalp topographies of the P45 and P300 across the rest condition and movement phases.

A repeated-measures ANOVA revealed a temporal modulation in P45 gating across movement phases (*F*_(3,99)_ = 3.35, *p* = 0.044, $\eta_{p}^{2}=0.092$). Post hoc *t* tests showed significantly smaller P45_diff_ around maximum speed than in the late post-max phase (*t*_(33)_ = −2.53, *p* = 0.049, *d_z_* = −0.43, 95% CI [−1.73– −0.19]), indicating reduced P45 gating around maximum speed, consistent with the psychophysical results. No significant differences were observed in P45_diff_ between the other movement phases (all *t*_(33)_ ≥ −2.41, *p* ≥ 0.066, *d_z_* ≥ −0.41; Fig. 4*B*). Lastly, a significant repeated-measures correlation between PSE_diff_ and P45_diff_ (*t*_(101)_ = 2.09, *p* = 0.039, *r* = 0.204, 95% CI [0.01–0.38]) revealed that stronger tactile suppression was associated with stronger P45 gating (Fig. 4*C*).

Taken together, these findings show that temporal changes in tactile suppression during reaching are associated with corresponding changes in early cortical processing. Perceptual reports, however, are shaped not only by early cortical processes but also by higher-order cognitive mechanisms such as stimulus evaluation and decision-making (Heekeren et al., 2008).

To test whether later cortical processing likewise exhibited dynamic changes across the movement, we also examined SEP gating in the P300 component at C3 (Fig. 5*B*,*C*), where the P300 showed its focal maximum in a previous study using the same stimulation protocol (Fuehrer et al., 2025). P300_diff_ was significantly greater than zero in all phases, reflecting consistent gating of the P300 throughout the movement: pre-max (*t*_(33)_ = 4.43, *p* < 0.001, *d_z_* = 0.76, 95% CI [1.24–3.33]), around max (*t*_(33)_ = 3.96, *p* < 0.001, *d_z_* = 0.68, 95% CI [1.30–4.05]), early post-max (*t*_(33)_ = 5.19, *p* < 0.001, *d_z_* = 0.89, 95% CI [1.73–3.97]), and late post-max (*t*_(33)_ = 2.95, *p* = 0.008, *d_z_* = 0.51, 95% CI [0.72–3.95]). In contrast to PSE_diff_ and P45_diff_, P300_diff_ did not significantly differ between movement phases (*F*_(2.26,74.71)_ = 0.68, *p* = 0.528, $\eta_{p}^{2}=0.02$), indicating no temporal modulation at this later stage of somatosensory processing. In sum, later P300 amplitudes were reduced during movement relative to rest, but this reduction did not mirror the phase-specific changes observed for tactile suppression and P45 gating.

## Discussion

We report temporal dynamics in tactile suppression during goal-directed reaching, with weaker suppression around maximum speed. This temporal modulation was paralleled in early somatosensory processing. As expected, P45 amplitudes were reduced during most movement phases relative to rest, reflecting early cortical gating (Rushton et al., 1981; Rossini et al., 1999). However, P45 responses near maximum speed were comparable to rest and larger than late in the movement. Reductions in P45 amplitude correlated with stronger tactile suppression, suggesting that changes in tactile sensitivity during movement were associated with early somatosensory processing. In contrast, P300 amplitudes were uniformly reduced during movement. This suggests that the temporal modulation reflects sensorimotor rather than cognitive processes. Our findings are consistent with reports of phase-dependent sensory modulation during goal-directed movements (Dimitriou et al., 2013; Česonis and Franklin, 2020; Wachsmann et al., 2025a). Similarly, a previous study using the same task reported a complete release from suppression in this movement phase, with tactile sensitivity around maximum speed matching sensitivity at rest (Voudouris and Fiehler, 2021). Although our results mirror those reported in that study, we still observed tactile suppression across all movement phases, including maximum speed. This discrepancy may reflect differences in psychophysical task demands: earlier work used a binary detection response immediately after movement offset (Voudouris and Fiehler, 2021), whereas our delayed comparison task introduced higher tactile working memory demands by requiring stimulus retention until several seconds after movement completion (Katus et al., 2015; Tamè and Holmes, 2016). Consistent with this, the P300, which has been linked to working memory updating and is modulated by memory-related task demands (Polich, 2007), was uniformly reduced across all movement phases, suggesting that late processing of the standard vibration may have been attenuated even at phases of preserved early processing. However, the P45 component, a temporally precise index of sensory encoding tied to low-level stimulus features such as intensity (Schröder et al., 2021; Fuehrer et al., 2025), was preserved around maximum speed. Thus, despite stronger overall perceptual suppression in our task, both studies converge on a transient release from suppression around maximum speed. These findings provide a crucial association between perceptual modulation during movement and changes in early sensory processing.

Our results align with optimal feedback control accounts, which propose a state-dependent modulation of feedback gains during movement (Todorov and Jordan, 2002; Liu and Todorov, 2007). Consistent with this, visuomotor gains peak mid-reach and decrease upon approaching the target (Dimitriou et al., 2013). Tactile signals contribute to the transport phase of reaching (Gentilucci et al., 1997), possibly by providing proprioceptive guidance (Aimonetti et al., 2007; Moscatelli et al., 2019). Thus, reduced tactile suppression around maximum speed may reflect a transient upregulation of somatosensory feedback during the transition from movement initiation to feedback-driven deceleration (Elliott et al., 2001). Alternatively, the effect could reflect stronger late suppression as the motor system shifts from position control to movement termination or anticipates target contact (Liu and Todorov, 2007; Fraser and Fiehler, 2018). Indeed, previous work on bimanual self-touch showed that tactile suppression on the resting target hand is minimal at the start of the movement and increases as contact with the other hand approaches, depending on the expectation of the touch (Cemeljic et al., 2025). Furthermore, temporal expectation of the standard vibration itself could influence the results, because the conditional probability of stimulation increased over the trial when no earlier stimulation had occurred. In this case, if a stimulus was not perceived during the later reaching phases, participants might have had higher expectations that a stimulus might arise. However, temporal expectations have been found to increase the just noticeable differences but not the PSE in a tactile discrimination task (Xiong et al., 2025). Because tactile suppression in the present study was quantified based on PSE shifts, temporal expectation is unlikely to account for the observed phase-dependent modulation of suppression. Temporal expectation could affect long-latency SEPs, while evidence for the influence on early SEPs such as the P45 is unclear (Lange and Röder, 2006; Ren et al., 2025). Critically, both contact expectancy and temporal expectation would predict a monotonic modulation over the course of the movement, whereas both tactile suppression and P45 gating were already pronounced early in the movement and transiently reduced around maximum speed, while P300 gating was uniform across the time course. This argues against a purely late suppression account and suggests selective preservation of early somatosensory processing when feedback demands are highest.

The mid-reach reduction in tactile suppression contrasts with reports of higher movement speed increasing suppression (Angel and Malenka, 1982; Cybulska-Klosowicz et al., 2011) and reducing cortical responses (Rushton et al., 1981; Chapman et al., 1988), perhaps due to stronger reafferent masking (Laskin and Spencer, 1979). However, those studies involved isolated joint movements with minimal precision demands. In contrast, suppression during complex movements requiring precision appears unaffected by increased reafferent input from stronger forces (Broda et al., 2020). In our somatosensory-guided task without visual feedback, tactile input may have become more relevant just before the deceleration phase. These findings indicate that suppression is not merely a function of movement parameters like speed but depends on the task relevance of somatosensory input. This aligns with previous evidence of modulations in short-latency SEPs by movement demands in balancing and precision tasks (Knecht et al., 1993; McIlroy et al., 2003; Wasaka et al., 2017). Similar phase-dependent modulation has been reported in locomotion (Chapin and Woodward, 1982; Duysens et al., 1990; Altenmüller et al., 1995; Mouchnino et al., 2015). Our results extend this work by showing that phase-dependent SEP modulation during somatosensory-guided reaching is associated with parallel behavioral changes in tactile sensitivity.

Unlike traditional SEP paradigms using transcutaneous nerve stimulation (Cruccu et al., 2008), we applied vibrations in the preferred range of Pacinian mechanoreceptors and rapidly adapting type II (RAII) fibers (Bolanowski et al., 1988). This allowed the examination of early cortical responses to naturalistic, low-threshold mechanoreceptor signals rather than synchronized mixed-fiber activation (Gardner et al., 1984). While the stimulation primarily engaged Pacinian mechanoreceptors, reaching likely generated input from a range of cutaneous and proprioceptive receptors. The P45 component arises mainly from S1 (Allison et al., 1992), which has been associated with early cutaneous–proprioceptive integration (Kim et al., 2015). The observed modulation may therefore reflect broader regulation of somatosensory input, beyond RAII-specific tuning.

Since movement-related gating of somatosensory responses has been documented at multiple stages of the ascending pathway, from first-order synapses in the spinal dorsal column to thalamic nuclei (Ghez and Pisa, 1972; Chapman et al., 1988; Seki and Fetz, 2012), the reductions in P45 amplitude observed here may reflect decreased afferent inflow from subcortical structures into S1, resulting in decreased activity at the cortical stage. At the spinal level, gating is mediated by presynaptic inhibition via local interneurons (Bui et al., 2015). In primates, this presynaptic inhibition is partly driven by descending pathways from the brainstem and cortex and emerges before EMG onset (Seki et al., 2003). Consistent with this, in the rodent whisker system, movement-related gating at the first synaptic stage is abolished when S1, S2, and posterior parietal cortex are lesioned, indicating top–down control of sensory gating already at the first synaptic stage (Chakrabarti and Schwarz, 2018). Converging evidence from humans likewise implicates S1, S2, posterior parietal cortex and the supplementary motor area in downregulating somatosensory perception during movement (Haggard and Whitford, 2004; Arikan et al., 2021). Although descending pathways are likely involved in regulating presynaptic inhibition, stronger gating at cortical than spinal levels in primates suggests additional corticocortical modulation (Seki and Fetz, 2012). For example, the motor cortex can recruit inhibitory neurons in the rodent auditory cortex to attenuate self-generated sounds (Schneider et al., 2018), illustrating how motor-related signals can engage local inhibitory circuits in the sensory cortex. Together, these findings support cortical control of tactile feedback gains, with descending pathways regulating afferent inflow and intracortical circuits modulating early cortical responses. The transient mid-reach release from P45 gating may therefore reflect reduced presynaptic inhibition along the subcortical pathway, decreased intracortical inhibition, or both. Resolving the precise origin of the observed modulation will require combined cortical and subcortical measures, including the N20 and earlier components evoked by electrical stimulation (Lei et al., 2018).

Subcortical and cortical gating mechanisms operate within a bidirectional sensorimotor control loop in which motor commands shape sensory inflow and somatosensory feedback influences motor output (Azim and Seki, 2019). Cutaneous and proprioceptive signals tune reflexes and shape ongoing movements by regulating motoneuron excitability via spinal interneuron circuits (Panek et al., 2014; Bui et al., 2015). Disrupting presynaptic inhibition of proprioceptive afferents impairs goal-directed reaching in rodents (Fink et al., 2014), whereas blocking cutaneous input increases hand path variability in grasping (Gentilucci et al., 1997), indicating that both excessive and absent somatosensory feedback compromise motor control. Together, these findings suggest that sensory gain is dynamically tuned to task demands. The modulation in gating observed in the early cortical responses here may reflect such adjustments of reafferent gain, which are proposed to suppress redundant somatosensory input while allowing behaviorally relevant feedback to influence motor circuits (Azim and Seki, 2019). Consequently, changes in perceived tactile intensity during movement may represent the behavioral expression of this distributed sensorimotor gating (McComas, 2016; Azim and Seki, 2019).

### Limitations

One limitation concerns potential overlap of SEPs with task-related events such as movement offset and target contact. This overlap would primarily affect the later P300, whereas the earlier P45 likely occurred during ongoing movement. Because these events were not systematically aligned with stimulation timing, any influence should be random and contribute as noise rather than as systematic bias. To mitigate these effects, we subtracted the EEG signal recorded during reaching alone from that recorded during reaching with stimulation. The subtracted signal was time-locked to the corresponding stimulation timing postmovement onset and therefore included the same unsystematic movement-related activity present during the stimulation epochs. This approach assumes an additive relationship between movement- and stimulation-related signals, although prior work has questioned this assumption (Quinn et al., 2014). Nevertheless, the resulting SEP waveforms matched canonical patterns reported for vibrations, including a frontoparietal dipole for the P45 and a centroparietal positivity for the P300 (Alouit et al., 2025; Fuehrer et al., 2025), supporting the validity of the method. Another limitation concerns the generalizability to visuomotor control. Although these findings align with work on visuomotor control showing increased reliance on sensory feedback mid-reach (Dimitriou et al., 2013), our task examined somatosensory-guided reaching without visual feedback. Future work should test whether similar temporal dynamics generalize to visually guided actions.

## Conclusion

In sum, tactile suppression is temporally tuned, with a distinct reduction around maximum speed. This modulation was accompanied by changes in early somatosensory processing, suggesting preserved processing when somatosensory input from the moving limb may be particularly relevant for the sensory guidance of the hand. This pattern suggests a flexible tuning of tactile suppression and SEP gating according to task demands. Higher-order processes appear to contribute to a general dampening of tactile perception but not to its fine-grained temporal tuning. This novel relation of psychophysical modulation to early neural markers suggests that tactile suppression is a flexible, context-sensitive mechanism coupled to sensorimotor demands in somatosensory-guided actions.

## Data Availability

Data have been deposited on the Open Science Framework: https://doi.org/10.17605/OSF.IO/5D8UZ
